# A Case-Control Study to Assess the Relationship between Poverty and Visual Impairment from Cataract in Kenya, the Philippines, and Bangladesh

**DOI:** 10.1371/journal.pmed.0050244

**Published:** 2008-12-16

**Authors:** Hannah Kuper, Sarah Polack, Cristina Eusebio, Wanjiku Mathenge, Zakia Wadud, Allen Foster

**Affiliations:** 1 International Centre for Eye Health, London School of Hygiene & Tropical Medicine, London, United Kingdom; 2 Cataract Foundation of the Philippines, Bacolod, Philippines; 3 Rift Valley Provincial Hospital, Nakuru, Kenya; 4 Child Sight Foundation, Dhaka, Bangladesh; University of New South Wales, Australia

## Abstract

**Background:**

The link between poverty and health is central to the Millennium Development Goals (MDGs). Poverty can be both a cause and consequence of poor health, but there are few epidemiological studies exploring this complex relationship. The aim of this study was to examine the association between visual impairment from cataract and poverty in adults in Kenya, Bangladesh, and the Philippines.

**Methods and Findings:**

A population-based case–control study was conducted in three countries during 2005–2006. Cases were persons aged 50 y or older and visually impaired due to cataract (visual acuity < 6/24 in the better eye). Controls were persons age- and sex-matched to the case participants with normal vision selected from the same cluster. Household expenditure was assessed through the collection of detailed consumption data, and asset ownership and self-rated wealth were also measured. In total, 596 cases and 535 controls were included in these analyses (Kenya 142 cases, 75 controls; Bangladesh 216 cases, 279 controls; Philippines 238 cases, 180 controls). Case participants were more likely to be in the lowest quartile of per capita expenditure (PCE) compared to controls in Kenya (odds ratio = 2.3, 95% confidence interval 0.9–5.5), Bangladesh (1.9, 1.1–3.2), and the Philippines (3.1, 1.7–5.7), and there was significant dose–response relationship across quartiles of PCE. These associations persisted after adjustment for self-rated health and social support indicators. A similar pattern was observed for the relationship between cataract visual impairment with asset ownership and self-rated wealth. There was no consistent pattern of association between PCE and level of visual impairment due to cataract, sex, or age among the three countries.

**Conclusions:**

Our data show that people with visual impairment due to cataract were poorer than those with normal sight in all three low-income countries studied. The MDGs are committed to the eradication of extreme poverty and provision of health care to poor people, and this study highlights the need for increased provision of cataract surgery to poor people, as they are particularly vulnerable to visual impairment from cataract.

## Abstract 2

Hannah Kuper and colleagues report a population-based case-control study conducted in three countries that found an association between poverty and visual impairment from cataract.

## Editors' Summary

**Background.:**

Globally, about 45 million people are blind. As with many other conditions, avoidable blindness (preventable or curable blindness) is a particular problem for people in developing countries—90% of blind people live in poor regions of the world. Although various infections and disorders can cause blindness, cataract is the most common cause. In cataract, which is responsible for half of all cases of blindness in the world, the lens of the eye gradually becomes cloudy. Because the lens focuses light to produce clear, sharp images, as cataract develops, vision becomes increasingly foggy or fuzzy, colors become less intense, and the ability to see shapes against a background declines. Eventually, vision may be lost completely. Cataract can be treated with an inexpensive, simple operation in which the cloudy lens is surgically removed and an artificial lens is inserted into the eye to restore vision. In developed countries, this operation is common and easily accessible but many poor countries lack the resources to provide the operation to everyone who needs it. In addition, blind people often cannot afford to travel to the hospitals where the operation, which also may come with a fee, is done.

**Why Was This Study Done?:**

Because blindness may reduce earning potential, many experts believe that poverty and blindness (and, more generally, poor health) are inextricably linked. People become ill more often in poor countries than in wealthy countries because they have insufficient food, live in substandard housing, and have limited access to health care, education, water, and sanitation. Once they are ill, their ability to earn money may be reduced, which increases their personal poverty and slows the economic development of the whole country. Because of this potential link between health and poverty, improvements in health are at the heart of the United Nations Millennium Development Goals, a set of eight goals established in 2000 with the primary aim of reducing world poverty. However, few studies have actually investigated the complex relationship between poverty and health. Here, the researchers investigate the association between visual impairment from cataract and poverty among adults living in three low-income countries.

**What Did the Researchers Do and Find?:**

The researchers identified nearly 600 people aged 50 y or more with severe cataract-induced visual impairment (“cases”) primarily through a survey of the population in Kenya, Bangladesh, and the Philippines. They matched each case to a normally sighted (“control”) person of similar age and sex living nearby. They then assessed a proxy for the income level, measured as “per capita expenditure” (PCE), of all the study participants (people with cataracts and controls) by collecting information about what their households consumed. The participants' housing conditions and other assets and their self-rated wealth were also measured. In all three countries, cases were more likely to be in the lowest quarter (quartile) of the range of PCEs for that country than controls. In the Philippines, for example, people with cataract-affected vision were three times more likely than normally sighted controls to have a PCE in the lowest quartile than in the highest quartile. The risk of cataract-related visual impairment increased as PCE decreased in all three countries. Similarly, severe cataract-induced visual impairment was more common in those who owned fewer assets and those with lower self-rated wealth. However, there was no consistent association between PCE and the level of cataract-induced visual impairment.

**What Do These Findings Mean?:**

These findings show that there is an association between visual impairment caused by cataract and poverty in Kenya, Bangladesh, and the Philippines. However, because the financial circumstances of the people in this study were assessed after cataracts had impaired their sight, this study does not prove that poverty is a cause of visual impairment. A causal connection between poverty and cataract can only be shown by determining the PCEs of normally sighted people and following them for several years to see who develops cataract. Nevertheless, by confirming an association between poverty and blindness, these findings highlight the need for increased provision of cataract surgery to poor people, particularly since cataract surgery has the potential to improve the quality of life for many people in developing countries at a relatively low cost.

**Additional Information.:**

Please access these Web sites via the online version of this summary at http://dx.doi.org/10.1371/journal.pmed.0050244.

- This study is further discussed in a *PLoS Medicine* Perspective by Susan Lewallen
- The MedlinePlus encyclopedia contains a page on cataract, and MedlinePlus also provides a list of links to further information about cataract  (in English and Spanish)
- VISION 2020, a global initiative for the elimination of avoidable blindness launched by the World Health Organization and the International Agency for the Prevention of Blindness, provides information in several languages about many causes of blindness, including cataract. It also has an article available for download on blindness, poverty, and development
- Information is available from the World Health Organization on health and the Millennium Development Goals (in English, French, and Spanish)
- The International Centre for Eye Health carries out research and education activities to improve eye health and eliminate avoidable blindness with a focus on populations with low incomes

## Introduction

Improvements in health are at the heart of the Millennium Development Goals, with the recognition that better health is central to the primary aim of reducing poverty as well as important in its own right. Empirical data are needed to back up this claim. Unravelling the relationship between blindness and poverty therefore has important implications, and may also be informative for the association between poverty and other disabilities.

Blindness is a common condition globally, affecting approximately 45 million people, and more than a third of blindness is caused by cataract [1,2]. Globally, the prevalence of blindness is five-fold higher in poor than rich countries [2]. Limited data show that within countries the poor are also more likely to be blind [3,4]. It is frequently asserted that blindness is both a cause and consequence of poverty, but there are few empirical data to support this claim. Poverty may cause cataract blindness, because access to cataract surgery is limited in low-income countries [5]. Furthermore, within poor countries some evidence suggests that lack of money is a major barrier to uptake of cataract surgery by individuals [6–8]. Blindness may also cause poverty, as the blind individual, or the household members who care for them, have a reduced earning potential [4,9]. This complex problem could have serious implications; estimates from The Gambia suggest that there is a substantial economic burden from lost productivity among blind people [10]. Therefore, blindness prevention may ultimately be cost saving [11]. Extrapolations on a global level indicate that a successful eye care programme could prevent more than 100 million cases of blindness between 2000 and 2020, and consequently save at least US$102 billion, which would otherwise be lost to reductions in productivity associated with blindness [12]. However, these estimates are based on extrapolations from limited data and were not based on individual-level data. It is also difficult to identify the component of productivity loss that is due to blindness, as this condition mainly affects older people, who may suffer from other comorbidities that restrict their employment opportunities or make them dependent on the care of others.

The Cataract Impact Study was undertaken to assess the relationship between cataract visual impairment and “economic poverty” and quality of life, and to estimate the impact of cataract surgery on these factors in three low-income countries. The aim of the current paper is to assess the association at baseline between visual impairment from cataract and household poverty (measured through consumption, asset ownership, and self-rated wealth) in a population-based case–control study in Kenya, the Philippines, and Bangladesh.

## Methods

### Setting

Case and control participants were recruited from Nakuru district, Kenya (January–February, 2005); Negros island (May–June, 2005) and Antique district (April–May, 2006), Philippines; and Satkhira district, Bangladesh (November–December, 2005).

### Selection of Cases and Controls

Persons with cataract visual impairment (cases) and persons without (controls) were primarily recruited through a population-based survey of adults aged ≥ 50 y [6–8]. Clusters of 50 people (regardless of visual impairment) aged ≥ 50 y were selected through probability-proportionate to size sampling, using either the census (Philippines and Bangladesh) or electoral role (Kenya) as the sampling frame. Households within clusters were selected through a modification of compact segment sampling, whereby a map was drawn of the enumeration area that was divided into segments, each including approximately 50 people aged ≥ 50 y, and one segment was chosen at random [13]. Households in the segment were included sequentially until 50 people aged ≥ 50 y were identified. The surveys included 3,503 (93% response rate) people aged ≥ 50 y in Kenya, 4,868 (92%) in Bangladesh, 2,774 (76%) in Negros, and 3,177 (83%) in Antique.

All people in the survey aged ≥ 50 y underwent visual acuity (VA) testing and ophthalmic examination. VA was measured in full daylight with available spectacle correction with a Snellen tumbling “E” chart using optotype size 6/18 (20/60) on one side and size 6/60 (20/200) on the other side at 6 or 3 metres. If the VA was <6/18 in either eye then pinhole vision was also measured. Participants with pinhole vision <6/18 but >6/60 in the better eye due to age-related cataract were given a second VA test using an “E” of size 6/24. The ophthalmologist examined all eyes with a presenting VA <6/18 with a torch (i.e., flashlight), direct ophthalmoscope, and/or portable slit lamp. The principal cause of blindness or visual impairment was recorded, according to the WHO convention in which the major cause is assigned to the primary disorder or, if there are two existing primary disorders, to the one that is easiest to treat [14].

Survey participants were eligible for inclusion as cases if they were aged ≥ 50 y with best corrected visual acuity <6/24 in the better eye due to cataract, as diagnosed by an ophthalmologist. All eligible cases identified from these surveys were invited to participate in the study. Participants were eligible to be controls if they were aged ≥ 50 y, did not have VA <6/24 in the better eye due to cataract and did not live in the same household as a case. During the survey a list was maintained of all eligible controls, by age group (50–54, 55–59, 60–64, 65–69, and >70) and sex. Whenever a case was identified, one age- and sex-matched control was randomly selected from the list for inclusion (or up to two controls in Bangladesh). If no matching eligible controls had been identified in that cluster at that stage of the survey, then the next eligible control in the cluster was recruited.

Because of logistical and time constraints, additional cases were also included through community-based case detection. In Kenya and Negros (Philippines), clusters were randomly selected through probability proportionate to size using the same cluster sampling procedure after completion of the population-based survey. Clusters were visited in advance and asked that all people ≥ 50 y with vision problems come to a central point on a specified day, and that a list be made of people unable to attend (e.g., due to blindness or other physical disability). After examining patients at the central point, the survey team then visited those unable to leave their houses. Any identified eligible cases that agreed to be part of the study were interviewed in their homes. In Bangladesh and Antique (Philippines), community case detection was carried out simultaneously with the survey by two of the four teams, so that controls were included for these cases. Within each cluster from the survey, one interviewer was asked to be taken to two community members aged ≥ 50 y with eye problems, living within the cluster boundaries but not from the segments selected for the survey. If VA was <6/24 with pinhole in the better eye, the ophthalmologist was called to carry out the full eye examination, and eligible cases were included in the study.

For the purposes of the present analyses, control individuals with any visual impairment (VA <6/18 in the better eye) were excluded (*n* = 14 in Kenya, *n* = 53 in Bangladesh, *n* = 24 in the Philippines). Case and control participants who were significantly communication impaired (e.g. deafness, dementia, or psychiatric disease) were excluded (fewer than five per country), and one case was excluded in the Philippines because of missing age data. One household had two eligible cases (Kenya), and one of these participants was excluded for the poverty analyses as poverty was assessed through household level indicators (see below).

In total, 147 cases (82 from the survey and 65 from case detection) and 79 controls were included in Kenya; 217 cases (162 from survey and 55 from case detection) and 280 controls in Bangladesh; and 238 cases (146 survey and 92 case detection) and 180 controls in the Philippines.

### Data Collection

All case and control participants were interviewed in their homes by trained interviewers in the local language. Each interview lasted approximately 1 h.

#### Measures of poverty.

Poverty was measured through (a) monthly per capita expenditure (PCE) to indicate consumption, (b) asset ownership, and (c) self-rated wealth. The economic part of the questionnaires was adapted through interviews, focus group discussions, and pilot testing in each country to ensure local relevance.

The person primarily responsible for household finances (which may have been the case/control or another household member) was interviewed to assess PCE and assets. PCE was measured using methods based on the World Bank's Living Standards Measurement Study [15]. Items were included on food (42–52 items per country), education (three items), health (five items), household expenses (nine items), and personal expenses (21 or 22 items). In total, 85 items were included in the questionnaire in Kenya, 90 in the Philippines, and 79 in Bangladesh. The informant was asked to recall the monetary value of food that was purchased, consumed from home production, or received as payment in kind or as gifts. Consumption was assessed over a 1-wk period for frequently consumed items, and this was scaled up to estimate monthly consumption. The amount consumed monthly was assessed for items that were consumed more rarely. Monthly rent was recorded among households who rented, and households who owned their property were asked to estimate the amount that they could charge in rent per month. The consumption on all items was summed to calculate total monthly household consumption, and this was converted to United States dollars (US$) at the 2005 exchange rate ($1 = 76 Kenya shillings, 64 Bangladesh taka, 55 Philippine pesos). Total monthly household consumption was divided by the number of household members to calculate monthly PCE for the household.

The household informant was also asked about the number and type of context-specific assets owned by the household, including different types of furniture, electrical equipment, cattle, and vehicles. Information was collected on household characteristics, including the building material of the floor, roof, and walls; type of toilet; and the number of rooms.

Self-rated wealth was assessed by asking the household informant to rank the household's wealth relative to others in the community on a scale from 1 (poorest) to 10 (richest).

#### Covariates.

Case and control individuals were interviewed about standard sociodemographic indicators, including household composition, education, and employment. Information was collected on vision-related quality of life using the World Health Organization Prevention of Blindness and Deafness 20-item Visual Functioning Questionnaire [16,17], and health-related quality of life was assessed using items from the European Quality of Life Questionnaire [18]. Detailed time-use data were collected using methods based on the World Bank's Living Standards Measurement Study [15].

### Training and Fieldwork

Interviewers were trained for 1 wk, including 2 d of pilot testing. Attempts were made to minimise measurement bias by emphasising the need for consistency in data collection among cases and controls. The questionnaires were translated into the local languages (three in Kenya, three in the Philippines, and one in Bangladesh) and back-translated by independent translators (one for each language) who were also asked to comment on appropriateness of language used for the target population. A review was held to discuss differences in translation and modify accordingly. The questionnaire was piloted in each setting and small modifications to wording of some items were made, where appropriate, to ensure local understanding. Teams were accompanied by a field supervisor at least 1 d per wk to ensure that high quality was maintained and interviews were observed randomly throughout the study.

### Statistical Analysis

Microsoft Access was used for data entry, and all data were double entered and validated. Analyses were undertaken in SAS version 8.2.

The mean and range of each expenditure item was calculated to assess whether answers were plausible, and to identify and exclude gross outliers (none identified). Rental equivalents were imputed based on household characteristics and non-rent expenditure for households where these estimates were missing or unreasonably low (< $1 per mo) (four in Kenya, three in Bangladesh, 18 in the Philippines). Total monthly household consumption was divided by the number of household members to calculate per capita household expenditure. Per capita household expenditure was divided into quartiles, separately for each country, based on the distribution of the data for the case and control participants combined. Households with incomplete expenditure data were excluded from analyses (five cases and four controls in Kenya; one case and one control in Bangladesh).

A relative index of household assets was derived using principal components analysis (PCA) to determine weights for a list of assets and wealth indicators [19]. Variables entered into the PCA included building materials of the house, ownership of ten household assets, animal ownership, and education of the head of the household. The derived index was divided into quartiles from poorest (lowest socioeconomic status [SES] index) to least poor (highest SES index). PCA analyses were undertaken separately for each country. The means of the poverty variables were first compared for cases recruited through the two different methods, and then from cases and controls using *t*-tests for continuous variables (e.g., PCE and assets). For categorical variables (e.g., household rank) we used the Mann-Whitney test and presented medians and interquartile ranges. PCE was highly skewed and therefore was log transformed for the *t*-tests. The two-way correlations were calculated between PCE, assets, and household rank, in turn.

Logistic regression analyses were undertaken separately for each country, assessing the association between case/control status and sociodemographic and poverty variables. Conditional logistic regression was not undertaken, since the matching was incomplete, so all analyses were adjusted for the matching variables (age, sex, and rural/urban location). Likelihood ratio tests were undertaken to assess the significance of adding covariates with more than two levels (e.g., age groups, self-rated health groups) to the model. Tests for trend were undertaken across quartiles of the poverty variables and assessed using the *p*-value for trend. Analyses were also conducted adjusting for the logistic regression analyses for poverty by social support indicators (marital status and household size) and self-rated health, since these variables may confound the association between cataract visual impairment and poverty. Analyses from the Philippines were also adjusted for study site, since data were obtained from two settings (Negros and Antique). An attempt was made to disentangle the relationship between poverty and cataract by stratifying the analyses by age, sex, and level of visual impairment among the cases.

### Ethical Approval

Informed signed or thumb-printed consent was obtained from all cases and controls. In Kenya and Bangladesh all cases were offered free cataract surgery at the local hospital, with free transport. In the Philippines, patients were referred for surgery, which was subsidised for patients who could not afford the fee. Ethical approval for this study was obtained from the ethics committees of the London School of Hygiene & Tropical Medicine, the Kenya Medical Research Institute, the Bangladesh Medical Research Council, and the University of St. La Salle, Bacolod, Philippines. This study complied with the guidelines of the Declaration of Helsinki.

## Results

### Sociodemographic Characteristics of Cases and Controls

Case and control participants were matched reasonably closely by sex and location. However, within the age category ≥ 70 y, cases tended to be older than the controls, so that cases were over-represented in the oldest age groups (75–79 and ≥ 80 y) compared to controls (Table 1). Cases were less likely to be married than controls, in Kenya (OR 0.6, 95% CI 0.3–1.1), Bangladesh (0.6, 0.4–1.0), and the Philippines (0.7, 0.4–1.0), although this only reached statistical significance in Bangladesh (*p* = 0.03). There was a strong protective effect of literacy and education on cataract in Bangladesh and Kenya that was not evident in the Philippines. Cases were substantially less likely to have a job other than working in the field compared to controls in all three countries. Cases reported significantly poorer self-rated health than controls—this pattern was particularly evident in the Philippines (OR for lowest versus highest quartile of self-rated health = 5.7, 95% CI 3.0–10.7) but also apparent in Kenya (2.6, 1.1–6.2) and Bangladesh (3.3, 2.1–5.3).

**Table 1:**
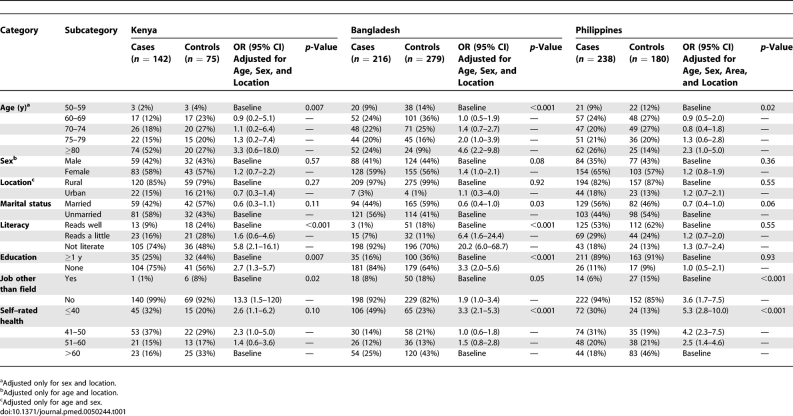
Sociodemographic Characteristics of Cases and Controls, in Kenya, Bangladesh, and the Philippines

### Summary Wealth Measures

**Figure 1:**
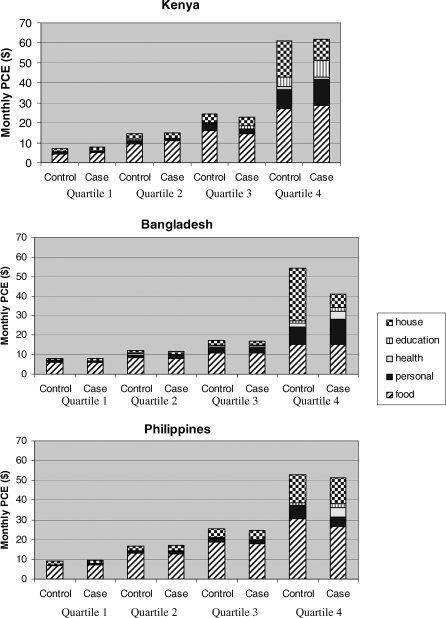
Per Capita Monthly Expenditure, by Quartile of Expenditure, for Cases and Controls in Kenya, Bangladesh, and the Philippines

All three settings were poor. The mean PCE was less than US$1 per person per day in all three settings: US$26.4 (standard deviation [SD] = US$34.9) in Kenya, US$21.7 (US$48.0) in Bangladesh and US$26.1 (US$23.5) in the Philippines. The biggest expense was food in all three settings, making up 55% of PCE in Kenya, 47% in Bangladesh, and 64% in the Philippines, followed by household expenses including rent (21% in Kenya, 28% Bangladesh, and 22% Philippines) (Figure 1). The majority of food consumption was from direct purchase (70% in Kenya, 75% in Bangladesh, and 77% in the Philippines) or home-grown production (24% in Kenya, 22% in Bangladesh, and 17% in the Philippines), and little was from gifts or payments.

An asset score was created through PCA in the three settings. The first principal component explained 22% of the variability in asset variables in Kenya, 25% in Bangladesh, and 24% in the Philippines. Self-perceived wealth of the household clustered around the average with a large proportion of households in Kenya (48%), Bangladesh (43%), and the Philippines (64%); households stating that they were ranked between 4 and 6, on a scale from 1 to 10, in terms of wealth in their community. The three measures of poverty were highly correlated, each showing significant correlation (*p* < 0.001) with the other measure.

### Economic and Household Characteristics of Cases and Controls

There were no significant differences in PCE, assets, or household rank between cases recruited through the population-based survey and those recruited through case detection, with the exception that the case-detection cases had lower household rank in Kenya (mean = 3.7 versus 3.1, *p* = 0.02). Consequently, cases recruited through the two methods were combined in the subsequent analyses.

Cases were poorer than controls, in all three settings according to all three poverty measurements (Table 2). The mean PCE was 20%–28% lower for members of households with a case than for control households, and this difference was highly significant in Bangladesh and the Philippines; for Kenya it was lower but did not reach significance (*p* = 0.07). The PCA score for assets was significantly lower among cases than controls in Kenya and Bangladesh, and it was lower in the Philippines although it did not reach significance (*p* = 0.06). Self-perceived wealth was significantly lower for households with a case compared to control households in Kenya (3.4 versus 4.5) and Bangladesh (3.9 versus 4.6), though not in the Philippines (4.1 versus 4.3).

**Table 2:**
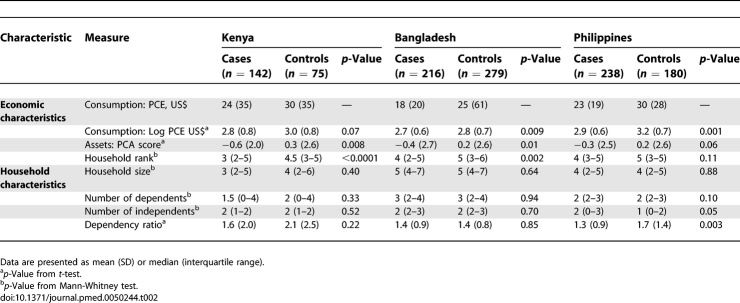
Household and Economic Characteristics, for Cases and Controls, in Kenya, Bangladesh, and the Philippines

There was no difference in the size of the households of cases and controls in any of the three settings. The ratio of dependents (i.e., household member aged <15 or ≥ 50 y) to independents (i.e., household member aged 15–50 y) was similar between cases and controls in Bangladesh (1.4 versus 1.4), but the dependency ratio was higher for controls than cases in Kenya (2.1 versus 1.6) and the Philippines (1.7 versus 1.3), due to the smaller number of people of working age.

### Patterns of Expenditure in Cases and Controls

Figure 1 shows the total PCE and the allocation of expenditure within quartiles of PCE for cases and controls. Monthly PCE was similar for cases and controls within each of the quartiles of expenditure. There was a gradual increase in PCE between the first three quartiles, and then a rapid increase between the third and the richest quartile. Within the first three quartiles of PCE the majority of expenditure was on food. Substantial expenditure on non-food items was observed only in the highest quartile of expenditure, where about half of expenditure was on non-food items. Similar patterns of PCE were observed for cases and controls in Kenya, Bangladesh, and the Philippines within each quartile of expenditure. These results demonstrate that cataract visual impairment was related to reduced PCE, but not allocation of expenditure.

### Multivariate Analyses of Poverty and Cataract Visual Impairment

Multivariate analyses showed that case participants were consistently poorer than controls in Kenya, Bangladesh, and the Philippines, using three different measures of poverty (Table 3). Cases were more likely than controls to be in the lowest quartile of PCE rather than the highest quartile in Kenya (OR 2.3, 95% CI 0.9–5.5), Bangladesh (1.9, 1.1–3.2) and the Philippines (3.1, 1.7–5.7). In all three settings these associations showed significant dose–response as assessed by the *p*-value for trend across the quartiles, with decreasing PCE related to case status and these relationships persisted after adjustment for self-rated health and social support indicators. A similar pattern was observed for the relationship between case–control status and asset ownership. Cases were significantly more likely to be in the lowest quartile of asset ownership rather than the highest quartile compared to controls in Kenya (3.7, 1.4–9.6), Bangladesh (2.6, 1.5–4.4), and the Philippines (2.1, 1.1–3.8). Cases were also significantly more likely to be in the lowest quartile of household rank rather than the highest, compared to controls in Kenya (3.5, 1.5–8.0), Bangladesh (2.7, 1.6–4.7) and the Philippines (2.3, 1.1–4.8). The associations with assets and household rank also showed a significant dose–response relationship, and the associations were largely unchanged after adjustment for self-rated health and social support indicators. In Kenya and Bangladesh the relationship between PCE and case status was somewhat weaker than for the other measures of poverty, while the reverse was true in the Philippines.

**Table 3:**
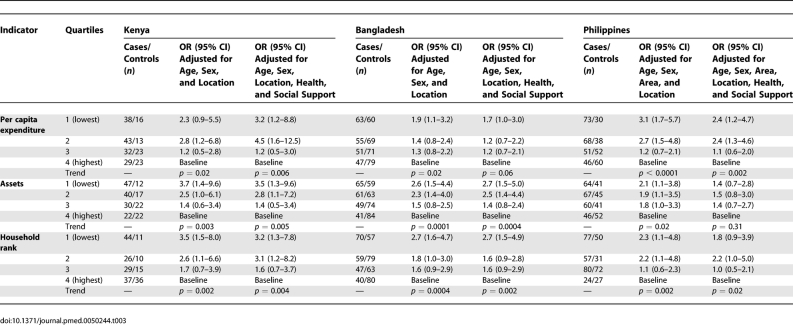
Association between Poverty and Cataract Visual Impairment in Kenya, Bangladesh, and the Philippines

Stratifying the association between PCE and cataract visual impairment by level of visual impairment showed an inconsistent pattern (Table 4). In Kenya, the association with low PCE was somewhat stronger comparing cataract blind cases to controls (OR 3.1, 95% CI 0.9–10.8) than comparing moderate visually impaired cases to controls (1.8, 0.6–5.4), while this pattern was reversed in Bangladesh (blind cases versus controls: 1.8, 1.0–3.4; moderately visually impaired cases versus controls: 3.1, 1.3–7.2). In the Philippines the association with low PCE was strongest comparing severely visually impaired cases to controls (5.9, 2.0–17.6). The association between cataract visual impairment and PCE was stronger among men than women in Bangladesh and the Philippines, while the reverse was true in Kenya (Table 5). In Kenya and the Philippines the strongest association between cataract and PCE was among people aged 70–79 y, while in Bangladesh the strongest effect was in people aged over 80 y. Stratifying the association between assets and household rank with cataract by level of visual impairment, sex, or age broadly repeated these findings, and generally supported the lack of consistent pattern (unpublished data).

**Table 4:**
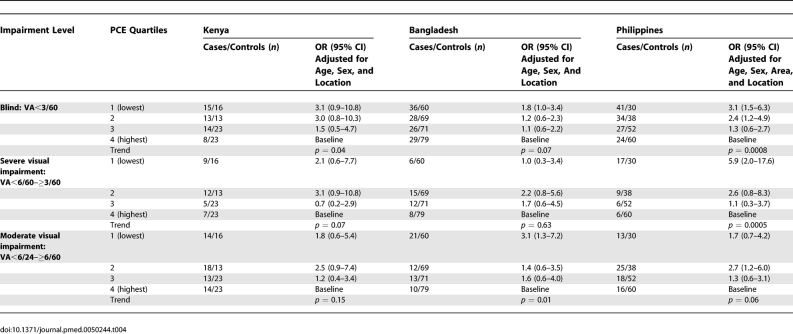
Association between Per Capita Expenditure and Cataract in Kenya, Bangladesh, and the Philippines, Stratified by Level of Visual Impairment

**Table 5:**
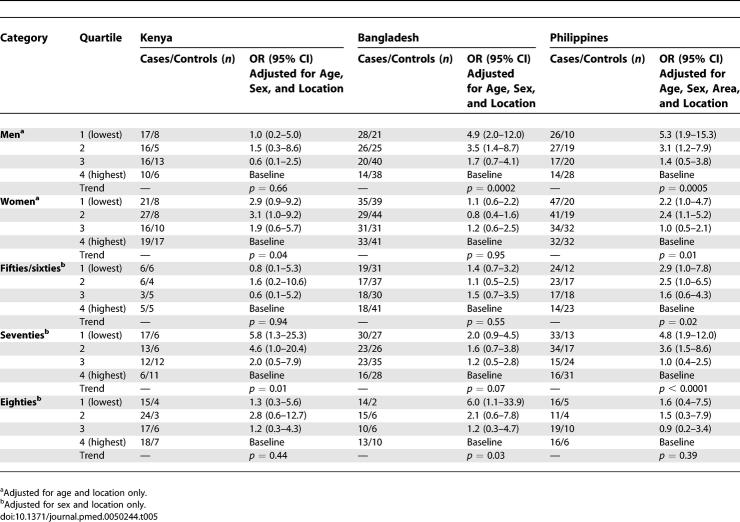
Association between Per Capita Expenditure and Cataract in Kenya, Bangladesh, and the Philippines, Stratified by Age and Sex

## Discussion

This large, multicentre population-based case–control study provides evidence that people with visual impairment from cataract are poorer than control participants with normal vision matched for age and sex. This pattern was evident whether poverty was measured in terms of PCE, assets, or self-rated wealth. Marital status seemed to be protective for cataract visual impairment, possibly indicating the role of social support in health-seeking behaviour. Reduced self-rated health was also strongly related to cataract visual impairment. This demonstrates the impact of poor vision on overall assessments of health and supports our previous finding of a relationship between cataract and quality of life [17].

Adjustment for marital status and self-rated health did not entirely explain the association between poverty and cataract visual impairment, suggesting that it operated through other pathways. Visual impairment could cause poverty through reduced employment opportunities. We might therefore expect to see a stronger relationship between cataract and poverty among the blind case participants who may have fewer employment opportunities than among those less impaired (i.e., moderate visual impairment). Poverty may also cause visual impairment through restricted access to cataract surgery. In this case we would expect to see a stronger relationship between poverty and less severely affected cases (i.e., moderate visual impairment), as poor families may allocate money for surgery on members who are blind from cataract, so that poverty mainly restricts access to surgery among people who are moderately visually impaired. The relationships that we observed between level of visual impairment and cataract were inconsistent across the three settings. Perhaps this shows that both pathways were operating or that the dynamics of the relationship between poverty and blindness vary in different settings. Levels of literacy and education were lower among cases than controls. These long-term indicators of disadvantage are unlikely to have changed after the onset of cataract. This observation provides some evidence that poverty preceded blindness in our study participants.

It is frequently asserted that blindness is both a cause and consequence of poverty, but there are few empirical data to support this claim. Globally, the prevalence of blindness is five-fold higher in poor than rich countries [2], and data from Pakistan and India suggest that within countries the poor are more likely to be blind [3, 4]. Some blinding conditions are a direct consequence of poverty, notably trachoma, which thrives in poor areas lacking water and sanitation [20]. Other blinding diseases clearly contribute to poverty, such as onchocerciasis, which results in the abandonment of the fertile areas near to the rivers where the disease vector thrives [9]. A larger literature shows that poor people are more likely to be ill or disabled than their richer compatriots, ranging from general disability in India, Bulgaria, and Ghana [21]; common mental disorders in Brazil, Chile, India, and Zimbabwe [22]; deafness in Brazil [23]; and tuberculosis in China [24]. There are also some exceptions such as a case-control study in Rwanda which failed to show an association between PCE and musculoskeletal impairment, perhaps because the population was almost universally poor [25].

Poverty may increase the incidence of disease, particularly preventable diseases such as tuberculosis. Poverty may also restrict access to appropriate health care and so prolong the duration of disease. A study in rural Tanzania showed that care-seeking behaviour for childhood illness is worse among poorer families than among the relatively rich families [26]. Another Tanzanian study found that people with higher levels of asset ownership were more likely to obtain antimalarials even though they were less likely to be parasitaemic [27]. With respect to cataract, there is little evidence that prevention is possible, and so the main pathway from poverty to blindness is likely to be through reduced access to cataract surgical services. High health care costs may also exacerbate poverty. A study in rural China showed that ill health increases medical expenditure significantly, which detracts from expenditure on food, education, investment in farming, and participation in social activities [28]. Inability to afford cataract surgery is cited as the major barrier to the uptake of surgery in the surveys conducted in Kenya, the Philippines, and Bangladesh [6–8]. This indicates that the cost of surgery is perceived as substantial by many households, notwithstanding the problems of assessing the complex issue of barriers in the absence of in-depth qualitative interviews. Consequently, there are lower rates of cataract surgery among the poor [3].

Poverty may also limit the employment opportunities of the person with disability or their household members. This pattern has been demonstrated for people with HIV in South Africa [29], tuberculosis in China [24], or disability in Sri Lanka [22]. An impact of blindness on reduced employment or income has been observed in Guinea [9] and India [4]. A belief that blindness reduces the employment opportunities of household members is widespread, but so far there is limited supportive evidence. There is a further complication to investigations of the relationship between cataract and poverty, as the individuals with cataract are likely to be elderly and facing multiple disabilities. Our study took account of the potential impact of multiple disabilities, as we adjusted for self-rated health, which is closely related to overall health, and this adjustment had no overall impact on our results [30].

### Study Strengths

This was a large population-based case–control study, conducted in three countries, allowing international comparisons. This was the first study, to our knowledge, to relate PCE to visual impairment. We also measured assets, which reflects long-term access to resources, and self-rated wealth. We used expenditure as a proxy for income, which has aided both academic and nonacademic investigations. As one example, the notorious Chicago gangster Al Capone managed to escape prosecution for smuggling, gambling, bootlegging, and murder for years, but was eventually convicted of tax evasion, because the jury was convinced that his exorbitant expenses on clothes, furnishing, foods, and gifts were inconsistent with his claim that he had no income. Expenditure often provides a better measure of poverty than income for a number of reasons. Income may be variable by season, whereas households attempt to smooth expenditure over the year. People are more comfortable sharing information about expenditure than income, and it may be a more meaningful measure than income in an agrarian society as it reflects what the household is able to command based on its current income, borrowing ability, or household savings [31]. PCE also has advantages over assets, as it may be more responsive to change, which will be important for the follow-up analyses of the study participants after they have undergone cataract surgery.

### Study Limitations

There are a number of limitations relating to the measurement of poverty in this study. Our analyses focus on monetary indicators of poverty, while we acknowledge that health, education, and housing are also important. We concede that it is difficult to measure expenditure accurately [32,33], but this also true for the measurement of diet and other variables, which is standard practise in many epidemiological studies. Furthermore, a large number of items were included in our measure of expenditure so that the measure was comprehensive [33]. Expenditure data were not validated through diaries or other means, although assets and self-rated wealth correlated highly with PCE. Other recent estimates of expenditure are not available from surveys conducted in these countries to allow comparison. The per capita estimates of monthly gross national income from the World Development Indicators database show somewhat higher estimates in Kenya (US$48) and Bangladesh (US$40) than our PCE derived estimates, and far higher estimates for the Philippines (US$108). This discrepancy may be reasonable, as the World Development Indicators reflect national averages, while we sampled the households with elderly people in poor regions of the country, many of whom were visually impaired from cataract. PCE was calculated simply by dividing the total household expenditure by the number of household members, without inclusion of economies of scale or equivalence scales. There is no widely accepted alternative to the simple equal-sharing convention, and the majority of expenditure was on food which does not allow for economies of scale. Furthermore, there were slightly fewer people of working age in the control households in Kenya and the Philippines, so adjustment for equivalence scores would be unlikely to explain the higher poverty among cases. The case and control households were of similar sizes in the three settings, so economies of scales are unlikely to have explained the differences.

There were a number of limitations relating to study design. Unfortunately, we did not record the exact numbers of cases and controls who refused to participate or were unable to communicate (believed to be fewer than five in each country), so the response rate is unknown, but was believed to be high. A variety of methods were used for case recruitment, as we were not able to obtain enough cases through the survey alone. However, cases recruited through the population-based survey and through case detection had similar poverty characteristics.

### Conclusions

Our data show that people with visual impairment due to cataract were poorer than controls in three low income countries, Bangladesh, Kenya, and the Philippines. The Millennium Development Goals are committed to the eradication of extreme poverty and provision of health care to poor people. This study confirms an association between poverty and blindness and highlights the need for increased provision of cataract surgery to poor people, particularly since cataract surgery is a highly cost-effective intervention in these settings [34].

## Abbreviations

CI: confidence interval
MDGs: Millennium Development Goals
OR: odds ratio
PCA: principal components analysis
PCE: per capita expenditure
SD: standard deviation
SES: socio-economic status
VA: visual acuity
